# Beyond eosinophil depletion: long-term clinical trajectory and durability of response to benralizumab in patients with severe eosinophilic asthma and CRSwNP: an 18-month real-world study (the BANDOR study)

**DOI:** 10.3389/fimmu.2026.1950211

**Published:** 2026-10-19

**Authors:** Emanuele Bizzi, Alessandro Maria Marra, Giuseppina Manzotti, Antonio Gidaro, Valeria Ortolani, Enrico Iemoli, Linda Borgonovo, Chiara Asperti, Stella Modica, Elisabetta Greco, Sara Rotunno, Mona-Rita Yacoub, Stefano Ministrini, Francesco Bini, Cristiano Germinario, Angela Mauro, Francesco Paciullo, Sarah Damanti, Francesca Casarin, Martina Sculco, Patrizia Rovere Querini

**Affiliations:** 1 Internal Medicine, Vita-Salute San Raffaele University, Milan, Italy; 2 Pneumology Unit, Azienda Socio Sanitaria Territoriale (ASST) Rhodense, Milan, Italy; 3 Servizio di Allergologia, Casa di Cura Beato Palazzolo, Bergamo, Italy; 4 Internal Medicine, Ospedale Luigi Sacco, Aziende Socio Sanitarie Territoriali (ASST) Fatebenefratelli-Sacco, Milan, Italy; 5 Allergology and Clinical Immunology, Luigi Sacco Hospital, University Hospital and Academic Centre, Milan, Italy; 6 Allergology and Immunology Unit, ASST GOM Niguarda, Milan, Italy; 7 Allergy, Immunology and Rheumatology, Tor Vergata University Hospital, Rome, Italy; 8 Department of Internal Medicine, Ospedale San Pietro, Rome, Italy; 9 Center for Molecular Cardiology, University of Zurich, Zurich, Switzerland; 10 Farmacia Marsico, Acquaviva delle Fonti, Bari, Italy; 11 Pediatric Rheumatology Unit, Department of Childhood and Developmental Medicine, ASST Fatebenefratelli-Sacco, Milan, Italy; 12 Department of Internal Medicine, Ospedale Fatebenefratelli e Oftalmico, Milan, Italy

**Keywords:** benralizumab, CRSwNP, FEV 1, severe asthma, SNOT 22

## Abstract

**Background:**

The management of patients with severe eosinophilic asthma and chronic rhinosinusitis with nasal polyps requires therapies capable of inducing stable remission. While benralizumab is an established treatment for severe asthma, randomized controlled trials in nasal polyposis have failed to reach primary clinical endpoints or showed inconsistent results. Consequently, there is a lack of evidence regarding its performance in clinical practice. The primary objective of this study was to evaluate the kinetics of response across different symptom domains and to identify predictors of clinical improvement in a Real-World setting in patients affected by severe eosinophilic asthma and chronic rhinosinusitis with nasal polyps.

**Methods:**

This retrospective observational study analyzed a cohort of patients with a United Airways Disease phenotype treated with benralizumab for a continuous period of 18 months. We mapped the longitudinal trajectory of specific SNOT 22 subdomains and lung function. Patients were stratified into early and late responders based on the timing of clinical improvement to identify baseline predictors of rapid versus gradual response.

**Results:**

Analysis revealed that SNOT 22 subdomains exhibit distinct recovery patterns. Nasal and physical symptoms showed a significant improvement within the first months of therapy despite the limited success reported in previous clinical trials. In contrast, functional and emotional domains required a longer period of treatment to reach a stable plateau. Early responders were characterized by a significantly higher baseline eosinophil count (median 885 vs 530 cells per microliter) and greater bronchial obstruction at baseline compared to late responders. Functional stability in FEV1 and a reduction in oral corticosteroid use were maintained throughout the observation.

**Conclusions:**

The BANDOR study demonstrates that benralizumab can induce durable clinical remission in a specific subset of patients who maintain treatment adherence. The speed of response is domain specific, with physical symptoms improving before emotional and functional ones. A high initial eosinophilic burden is the primary determinant of a rapid response. These findings suggest that while randomized trials showed limited efficacy, Real-World data identifies a high disease burden phenotype that derives significant long term benefit from receptor targeted eosinophil depletion.

## 1 Introduction

Severe eosinophilic asthma (SEA) and chronic rhinosinusitis with nasal polyposis (CRSwNP) are frequently coexisting manifestations of a single, systemic inflammatory process affecting the respiratory tract (1–3). This clinical intersection is best understood through the United Airways Disease (UAD) hypothesis, where upper and lower airways form a continuous functional and eventually pathological unit and inflammation in the nasal mucosa and the bronchial tree may represent local expressions of a common mucosal susceptibility where an exacerbation in one compartment often leads to a following worsening of the other (4–8).

At the molecular heart of both SEA and CRSwNP lies Type 2 inflammation (9–11). Eosinophils act as the primary effector cells in this inflammatory cascade (1, 2, 12, 13). In the lower airways, eosinophilic infiltration leads to bronchial hyperresponsiveness, epithelial damage and airway remodeling, manifesting as severe, steroid-resistant asthma. In the upper airways, these same cells drive the formation of nasal polyps, benign edematous growths of the nasal mucosa, which cause chronic obstruction, anosmia and significant deterioration of patients’ quality of life. The presence of high tissue and peripheral eosinophilia is a hallmark of more aggressive disease phenotypes and determines a higher risk of recurrence after surgical intervention.

Benralizumab is a humanized afucosylated monoclonal antibody that represents a unique therapeutic approach in this landscape. Benralizumab targets the alpha subunit of the IL-5 receptor (IL-5Rα) directly on the surface of eosinophils and basophils (14–18) and blocks IL-5 signaling and triggers antibody-dependent cell-mediated cytotoxicity (ADCC), leading to a rapid, nearly complete and sustained depletion of eosinophils in the blood, bone marrow, and, crucially, the target tissues (14).

While benralizumab has demonstrated robust clinical efficacy in reducing exacerbations and improving lung function in SEA, its role in CRSwNP has been more complex. Two major Phase III clinical trials were designed to evaluate benralizumab specifically for nasal polyposis (19–21). Although these studies provided evidence of clinical benefit, they did not consistently reach all primary endpoints and showed results that were less pronounced than those seen with other anti-T2 biologics. This discrepancy suggests that the one-size-fits-all approach may not be suitable and that the clinical response might depend on specific patient characteristics or the presence of comorbid asthma.

While the systemic depletion of eosinophils is a potent mechanism, the heterogeneity of Type 2 inflammation suggests that the therapeutic impact on nasal polyposis may be modulated by specific clinical and biological endotypes (4).

## 2 Rationale of the study and objectives

The primary BANDOR’s rationale is to evaluate the Real-World effectiveness of benralizumab in a dedicated cohort of patients suffering from the dual burden of SEA and comorbid CRSwNP. The complexity of managing the UAD patient lies in the significant variability of their clinical trajectory. While anti-IL-5Rα therapy is effective on asthma, we are still faced with the challenge of why some patients experience a near total resolution of their sinonasal symptoms almost immediately, while others show a more gradual or partial improvement (4, 20, 22). This gap in our understanding highlights that T2-high inflammation is not a monolithic entity, but a spectrum (22–24).

The BANDOR study was designed not merely to corroborate the established efficacy of benralizumab in SEA, but to interrogate the specific clinical signatures that define who and when of therapeutic success. Strong scientific basis suggest that the answers to these discrepancies are embedded within baseline clinical profiles (25–30). By examining the intersection of demographic variables, such as the age at asthma onset, and biological markers, including the magnitude of peripheral eosinophilia and IgE sensitization, this study seeks to map the specific phenotypes most receptive to IL-5Rα inhibition.

Furthermore, by correlating objective lung function parameters, such as forced expiratory volume in one second (FEV1), with validated patient-reported outcomes like the SNOT-22 and ACT scores, the study aims to establish a robust link between physiological data and perceived quality of life.

In particular, as a first objective, by adopting a longitudinal perspective, this study sought to characterize the dynamics and trajectories of therapeutic response across both the upper and lower respiratory compartments in patients with SEA and CRSwNP, verifying if the targeted depletion of IL-5Ra positive cells translates into a synchronized clinical improvement in these interconnected districts.

A key secondary objective was to move beyond the simple assessment of global efficacy, by identifying specific baseline clinical and functional determinants that could act as reliable predictors of a rapid treatment response. In particular, we focused on the identification of early responders, patients achieving the Minimal Clinically Important Difference (MCID) for the SNOT-22 score within the first six months, that is defined as a reduction of at least 9 points from the baseline score (recognized in the literature as the smallest change in the SNOT-22 score that patients perceive as a meaningful improvement in their quality of life (31). Through this approach, the study aimed to determine whether initial functional impairment, measured via FEV1 thresholds, or specific symptomatic clusters, identified through SNOT-22 subdomains, could serve as phenotypic markers of heightened sensitivity to benralizumab.

Ultimately, BANDOR study aimed to contribute to the evolving framework of Precision Medicine within the UAD paradigm.

## 3 Materials and methods

### 3.1 Study design and ethical framework

The BANDOR study was designed as a national, multicenter, retrospective observational investigation. The study architecture was specifically developed to evaluate the Real-World clinical effectiveness of benralizumab in a highly characterized cohort of patients suffering from the clinical overlap of SEA and CRSwNP.

The study was coordinated by the IRCCS Ospedale San Raffaele in Milan and conducted across a network of specialized immunology, allergy and respiratory medicine centers throughout Italy. This multicenter approach was intentionally chosen to ensure a diverse patient population, to enhance the external validity and generalizability of the findings beyond a single institution setting.

Methodologically, the study employed a longitudinal before-after retrospective design covering a total observation period of 18 months. Clinical, functional and biological data were systematically extracted from medical records at five distinct time points:

- Baseline (T0): representing the period immediately preceding the first administration of benralizumab.
- Follow-up intervals: data were subsequently collected at 3 months (T3), 6 months (T6), 12 months (T12), and 18 months (T18) of continuous therapy.

This extended 18 months window and the inclusion of multiple intermediate time points were pivotal to capture the kinetics of the therapeutic response. This allowed for the assessment of early therapeutic stabilization, the achievement of the MCID in sinonasal symptoms, and, crucially, the sustainability of the clinical benefit over the long term.

Ethical oversight was a cornerstone of the study protocol. The investigation was conducted in full accordance with the principles of the Declaration of Helsinki and the standards of Good Clinical Practice (GCP). The study protocol was reviewed and formally approved by the Ethical Committee of the IRCCS Ospedale San Raffaele (the coordinating center) and subsequently by the Institutional Review Boards (IRBs) of all participating satellite sites.

The retrospective nature of the study allowed for the observation of Real-Life clinical practice, capturing the outcomes of patients treated according to standard therapeutic indications without the restrictive selection biases often encountered in randomized controlled trials (RCTs). This design was aimed at achieving the study’s primary goal: identifying the Real-World clinical determinants that characterize early and late responders to IL-5Rα targeted therapy.

### 3.2 Patient selection and inclusion/exclusion criteria

The study population was derived from a systematic screening of medical databases at the participating specialist centers. Eligible participants were adult patients (aged 18 years or older) characterized by a dual diagnosis of SEA and CRSwNP, whose clinical management required the initiation of benralizumab according to current international guidelines and local reimbursement criteria.

To ensure high diagnostic accuracy, the inclusion framework was structured as follows:

- Asthma severity and phenotype: all patients were required to have a documented history of severe eosinophilic asthma. This was defined by the necessity for high-dose inhalatory corticosteroids (ICS) combined with a second controller (such as long-acting beta-agonists or long-acting muscarinic antagonists) or maintenance systemic corticosteroids to prevent the disease from becoming uncontrolled, or which remained uncontrolled despite this therapy. The eosinophilic endotype was confirmed by a history of elevated blood eosinophil counts prior to the initiation of any biologic intervention.
- Sinonasal characterization: the presence of CRSwNP was strictly validated according to the EPOS (European Position Paper on Rhinosinusitis and Nasal Polyps) criteria (32). This involved the presence of persistent sinonasal symptoms (nasal obstruction, hyposmia/anosmia, and rhinorrhea) for more than 12 weeks, coupled with direct visualization of polyps via nasal endoscopy or evidence of mucosal opacification within the paranasal sinuses on CT imaging.
- Therapeutic continuity: only patients who had remained on benralizumab for a minimum of 18 months were included in the final analysis. This duration was considered necessary to allow for the stabilization of the therapeutic effect and to permit a meaningful assessment of the changes in both bronchial and nasal parameters. To evaluate the long-term trajectory and durability of response, the analysis was restricted to a per-protocol population comprising exclusively patients who maintained continuous adherence to benralizumab for the entire 18-month observation window. Consequently, patients who discontinued therapy or were lost to follow-up prior to 18 months due to early lack of efficacy or other causes were not included in this final outcome analysis, which specifically focuses on the persistent responder profile.

To minimize confounding variables and ensure that the observed outcomes were directly attributable to benralizumab, a rigorous set of exclusion criteria was applied. Individuals were excluded if they:

1. Had been treated with other monoclonal antibodies targeting the T2 pathway within the 6 months preceding the baseline, thereby avoiding potential carry-over effects.
2. Presented with alternative pulmonary pathologies, such as cystic fibrosis, interstitial lung disease or active tuberculosis, which could interfere with the interpretation of FEV1 and respiratory symptom scores.
3. Demonstrated a history of poor adherence to their baseline controller therapies or lacked sufficient clinical documentation to verify their T0 status.
4. Were pregnant or breastfeeding at the time of treatment initiation.

Consequently, the study design focuses on a per-protocol maintenance cohort of persistent responders who completed the full 18-month observation period, excluding early treatment failures or patients who discontinued therapy.

A total of 54 patients were initially screened across the participating centers for this retrospective evaluation. Of these, 13 patients were excluded or discontinued therapy prior to the 18-month mark (6 due to lack of clinical efficacy within the first 6 months, 4 due to loss to follow-up, and 3 due to adverse events or administrative reasons), resulting in a final analysis cohort of 41 patients who completed the continuous 18-month treatment period.

### 3.3 Treatment protocol and data collection

All patients enrolled in the study received benralizumab via subcutaneous injection at the manufacturer’s recommended dosage of 30 mg. The therapeutic regimen followed the standardized induction and maintenance schedule for SEA (15). Throughout the observation period, the administration of benralizumab was integrated into the patients’ routine clinical care, ensuring that the results reflect Real-World clinical practice rather than a controlled experimental environment.

Data collection was executed through a rigorous retrospective review of electronic and paper-based medical records across all participating centers. To ensure data consistency, a standardized Case Report Form (CRF) was utilized to capture a comprehensive array of variables at two predefined milestones: Baseline (T0), immediately prior to the first dose, and Follow-up, occurring after 3, 6, 12 and 18 months of continuous therapy.

The data extraction process focused on four primary clinical domains:

- Anthropometric and clinical history: detailed records were compiled regarding age, sex, body mass index (BMI), and smoking status (categorized as never, former, or current smokers). Particular attention was paid to the age at asthma onset, a variable hypothesized to be a key determinant in treatment response.
- Biological and inflammatory profiling: peripheral blood samples were analyzed to determine absolute eosinophil counts (cells/µL). Additionally, total serum IgE levels (kU/L) and fractional exhaled nitric oxide (FeNo) measurements were recorded to characterize the underlying Type 2 inflammatory landscape.
- Respiratory functionality: objective assessment of lung function was performed using spirometry. The FEV1 was recorded both in absolute terms (Liters) and as a percentage of the predicted value, providing a standardized measure of bronchial obstruction.
- Symptom burden and quality of life: the clinical impact of the disease was quantified using validated, patient-reported outcome measures. Sinonasal symptom severity was assessed via the SNOT-22, which was further dissected into its five specific sub-domains: Nasal, Ear/Facial, Sleep, Function, and Emotion. Asthma control was simultaneously evaluated using the ACT score.

To maintain high data quality, all entries were cross-referenced for accuracy, and any patients with significant missing data at either T0 or follow-ups were excluded from the final analysis. This multidimensional data collection strategy was designed to provide a holistic view of the UAD response, bridging the gap between biological markers and the patient’s subjective clinical experience.

While baseline nasal endoscopy and paranasal sinus CT scans were systematically performed for initial diagnostic and EPOS staging purposes, standardized serial quantitative measures such as the Nasal Polyp Score (NPS) or follow-up Lund-Mackay CT scores were only retrospectively available for a minor subset of patients and lacked uniform timing across all longitudinal intervals, precluding a robust formal quantitative analysis of anatomical regression.

### 3.4 Definition of clinical response and identification of predictors

The primary metric for defining clinical response was the change in the SNOT-22 total score from baseline (T0) to each follow-up time.

To ensure that the observed changes translated into a perceptible benefit for the patient, the study adopted the Minimal Clinically Important Difference (MCID) as the threshold for response. The clinical effectiveness of Benralizumab on sinonasal symptoms was evaluated using the SNOT-22. To determine the clinical relevance of the observed changes, we adopted a 9-point reduction as the MCID (31). By utilizing this rigorous and conservative cut-off, we ensured that the reported improvements in the BANDOR study were not only statistically significant but also clinically impactful, reflecting a relevant reduction in the symptomatic burden for patients suffering from comorbid CRSwNP. Based on this criterion, the study population was stratified into two distinct cohorts:

1. Early responders: patients who achieved a decrease of ≥9 points in their SNOT-22 total score within the first 3 or 6 months of benralizumab therapy.
2. Late/suboptimal responders: patients who exhibited a more protracted trajectory, achieving the MCID at later time points or demonstrating a partial symptomatic response. As this study evaluated a per-protocol maintenance cohort completing 18 months of continuous therapy, true early discontinuation due to absolute non-response was not captured.

Beyond this primary categorization, the study investigated the “Determinants Of Response”, the specific baseline features that could distinguish these two groups before the initiation of therapy. The analysis evaluated several key clinical and biological dimensions as potential predictors:

- Anamnestic phenotyping: the age at asthma onset was scrutinized as a potential marker of disease endotype, evaluating whether early onset versus late onset disease influenced the magnitude of the response to IL-5Rα inhibition.
- Inflammatory load: baseline peripheral blood eosinophil counts were analyzed to determine if a specific threshold of systemic eosinophilia correlated with a more rapid or profound clinical improvement.
- Functional thresholds: initial respiratory capacity, measured via FEV1 percentage and absolute values, was examined to assess whether the severity of lower airway obstruction at baseline served as a limiting or facilitating factor for upper airway recovery.

Furthermore, to evaluate the multidimensional impact of the disease on patients’ quality of life, the baseline SNOT-22 subdomains were analyzed as potential determinants of clinical response. Specifically, we aimed to determine whether a higher symptom burden in specific clusters at baseline could identify early responders, thereby acting as phenotypic markers associated with rapid upper airway recovery.

This methodological approach allowed for a comparison between responders and late/non-responders, changing the usually applied focus of a general assessment of efficacy to the identification of a specific clinical signature, capable of predicting high probability success in the management of the UAD.

### 3.5 Statistical analysis

Continuous variables were tested for normality using the Shapiro-Wilk test. Normally distributed variables are presented as means/standard deviations (SD), while non-normally distributed variables are expressed as medians with interquartile ranges (IQR). Categorical variables are reported as counts and percentages. For longitudinal evaluations across the five assessment time points (T0, T3, T6, T12, and T18), repeated-measures ANOVA (with Tukey’s *post-hoc* test) or the Friedman test (with Dunn’s *post-hoc* correction) was applied depending on data normality. For between-group comparisons (early versus late/suboptimal responders), continuous variables were compared using Student’s t-test or the Mann-Whitney U test, whereas categorical variables were analyzed using Pearson’s Chi-square test or Fisher’s exact test. A two-sided p-value of $< 0.05$ was considered statistically significant. All statistical analyses were performed using standard software packages (SPSS version 26.0 or R).

## 4 Results

### 4.1 Baseline demographic and clinical characteristics

The study population comprised a total of 41 patients, whose baseline characteristics underscored a cohort suffering from a high burden and relapsing form of UAD. The demographic distribution was balanced, consisting of 20 females (48.8%) and 21 males (51.2%), with a median age of 50 years (IQR: 41–59) at the time of inclusion. This age profile is consistent with the typical presentation of adult onset T2-high airway disease.

A detailed analysis of the patients’ respiratory history revealed a median age at asthma onset of 30 years (IQR: 25–40). This timing is of particular clinical interest, as it points toward a predominantly adult onset phenotype, which is frequently associated with a more aggressive disease course and a higher prevalence of comorbid nasal polyposis (28, 33–35). From a lifestyle perspective, the cohort included 8 active smokers (19.5%), with the majority of the remaining patients being former smokers (87.5% of the non-active group) who had ceased smoking for a median of 24 months prior to baseline.

The systemic and localized inflammatory profiling at T0 confirmed a robust T2 endotype across the cohort. Patients exhibited a relevant systemic eosinophilia, with a median peripheral blood eosinophil count of 820 cells/µL (IQR: 515–1235). This was complemented by signs of systemic atopy and local airway inflammation, evidenced by a median total serum IgE of 145 kU/L (IQR: 71.5–445) and a median FeNO level of 31 ppb (IQR: 26–64). These markers collectively indicate that the study population at T0 was significantly inflamed and symptomatic despite being under conventional inhaled or systemic corticosteroid regimens.

The clinical complexity was further exacerbated by a high prevalence of comorbidities, which often act as complicating factors in the management of SEA and CRSwNP. gastroesophageal reflux disease (GERD) was the most frequent comorbidity, identified in 36.6% of the participants. Other significant clinical features included obesity (22.0%), bronchiectasis (14.6%) and a history of autoimmunity (19.5%). Notably, 22.0% of the patients had already experienced adverse events related to chronic OCS use, highlighting the urgent clinical need for OCS sparing biological alternatives in this group (36).

Functional and symptomatic assessments at baseline provided a clear and diffuse condition of uncontrolled disease. Lung function was markedly compromised, as shown by a median FEV1 of 2.52 L (IQR: 1.86–3.15), representing only 65% of the predicted value (IQR: 50.5–80.5). This bronchial impairment was reflected in a poor quality of life and inadequate symptom control, with a median ACT score of 13 (IQR: 10–16.5), a value significantly below the threshold of 20, that denotes clinical control.

The upper airway burden was even more pronounced. The median total SNOT-22 score reached 72 points (IQR: 58.5–82.5), indicating a severe impact on quality of life. When examining the specific subdomains of the SNOT-22, the Nasal domain exhibited the highest median score (21), followed by Function (13) and Sleep (12). This distribution emphasizes that the sinonasal involvement was not merely a localized anatomical issue, but a systemic burden affecting sleep architecture and overall functional capacity, justifying the multifaceted UAD approach of the BANDOR study. (See **Tables 1**, **2** for detailed results).

**Table 1:** Baseline demographic, clinical, biological, and functional characteristics of the study cohort and comparison between early responders and late/suboptimal responders.

Variable	Total cohort (n = 41)	Early responders (n = 30)	Late/suboptimal responders (n = 11)	p-value
**Age (years), median [IQR]**	50.0 [41.0–59.0]	49.0 [40.0–58.0]	52.0 [43.0–61.0]	0.420
**Sex (Female), n (%)**	20 (48.8%)	14 (46.7%)	6 (54.5%)	0.670
**Age at asthma onset (years), median [IQR]**	30.0 [25.0–40.0]	34.0 [25.0–40.0]	26.0 [25.0–30.0]	0.008
**Smoking status, n (%)**				0.810
- Never smoker	8 (19.5%)	6 (20.0%)	2 (18.2%)	
- Former/Active smoker	33 (80.5%)	24 (80.0%)	9 (81.8%)	
**Blood eosinophils (cells/µL), median [IQR]**	820.0 [515.0–1235.0]	885.0 [660.0–1420.0]	530.0 [360.0–1100.0]	< 0.001
**Total serum IgE (kU/L), median [IQR]**	145.0 [71.5–445.0]	150.0 [75.0–460.0]	130.0 [65.0–390.0]	0.550
**FeNO (ppb), median [IQR]**	31.0 [26.0–64.0]	34.0 [28.0–68.0]	28.0 [22.0–52.0]	0.210
**Baseline FEV1 (L), median [IQR]**	2.52 [1.86–3.15]	2.35 [1.75–3.00]	2.80 [2.10–3.40]	0.045
**Baseline FEV1 (% predicted), median [IQR]**	65.0 [50.5–80.5]	57.5 [44.0–70.0]	79.0 [60.0–90.0]	< 0.001
**Baseline ACT score, median [IQR]**	13.0 [10.0–16.5]	12.5 [9.5–16.0]	14.0 [11.0–18.0]	0.310
**Baseline total SNOT-22, median [IQR]**	72.0 [58.5–82.5]	75.0 [62.0–85.0]	66.0 [52.0–78.0]	0.120
- Nasal subdomain	23.0 [18.5–29.5]	23.0 [18.0–39.0]	17.0 [12.0–22.0]	0.015
- Ear/Facial subdomain	9.0 [6.0–13.0]	11.0 [8.0–15.0]	7.0 [4.0–10.0]	0.020
- Sleep subdomain	13.0 [9.0–16.0]	14.0 [10.0–16.0]	12.0 [8.0–15.0]	0.400
- Function subdomain	14.0 [10.0–18.0]	15.0 [11.0–18.0]	13.0 [9.0–16.0]	0.350
- Emotion subdomain	10.0 [7.0–14.0]	11.0 [8.0–14.0]	9.0 [6.0–13.0]	0.480
**Comorbidities, n (%)**				
- GERD	15 (36.6%)	11 (36.7%)	4 (36.4%)	0.980
- Obesity (BMI $\ge 30$)	9 (22.0%)	6 (20.0%)	3 (27.3%)	0.630
- Bronchiectasis	6 (14.6%)	4 (13.3%)	2 (18.2%)	0.700
- History of OCS-related adverse events	9 (22.0%)	7 (23.3%)	2 (18.2%)	0.740

( values are expressed as median and interquartile range [IQR] for continuous variables, and counts with percentages for categorical variables. Comparisons between early and late/suboptimal responders were performed using Mann-Whitney U test or Fisher’s exact test, as appropriate).

Bold values indicate statistically significant p-values (p < 0.05).

**Table 2:** Longitudinal evolution of clinical, functional, and biological endpoints across the 18-month observation period (total cohort, n = 41).

Endpoint	Baseline (T0)	3 months (T3)	6 months (T6)	12 months (T12)	18 months (T18)	P-value (overall)
**Total SNOT-22 score, median [IQR]**	72.0 [58.5–82.5]	34.0 [21.0–55.0]	26.0 [18.0–41.5]	23.0 [11.5–39.0]	22.0 [12.0–37.0]	< 0.001
- Nasal subdomain	23.0 [18.5–29.5]	11.0 [5.5–17.0]	9.0 [4.0–14.0]	8.0 [3.0–12.0]	7.0 [3.0–11.0]	< 0.001
- Ear/Facial subdomain	9.0 [6.0–13.0]	4.0 [2.0–7.0]	3.0 [1.0–6.0]	2.0 [1.0–5.0]	2.0 [0.0–4.0]	< 0.001
- Sleep subdomain	13.0 [9.0–16.0]	6.0 [3.0–10.0]	4.0 [2.0–8.0]	4.0 [1.0–7.0]	3.5 [1.0–6.0]	< 0.001
- Function subdomain	14.0 [10.0–18.0]	7.0 [4.0–12.0]	5.0 [2.0–9.0]	4.5 [2.0–8.0]	4.0 [1.0–7.0]	< 0.001
- Emotion subdomain	10.0 [7.0–14.0]	5.0 [2.0–9.0]	4.0 [1.5–7.0]	3.0 [1.0–6.0]	3.0 [1.0–5.0]	< 0.001
**ACT score, median [IQR]**	13.0 [10.0–16.5]	22.0 [19.0–24.0]	23.0 [18.5–24.0]	24.0 [21.0–25.0]	23.5 [20.2–25.0]	< 0.001
**FEV1 (L), median [IQR]**	2.52 [1.86–3.15]	2.68 [2.00–3.30]	2.80 [2.10–3.45]	2.82 [2.15–3.50]	2.85 [2.20–3.55]	< 0.001
**FEV1 (% predicted), median [IQR]**	65.0 [50.5–80.5]	71.0 [55.0–84.0]	75.0 [60.0–86.0]	76.0 [61.0–86.5]	77.0 [62.0–87.0]	< 0.001
**Blood eosinophils (cells/µL), median [IQR]**	820.0 [515.0–1235.0]	0.0 [0.0–10.0]	0.0 [0.0–0.0]	0.0 [0.0–0.0]	0.0 [0.0–0.0]	< 0.001

( values are expressed as median and interquartile range [IQR]. Longitudinal comparisons across the five time points were evaluated using the Friedman test with Dunn’s post-hoc correction).

Bold values indicate statistically significant p-values (p < 0.05).

### 4.2 Biological response and the kinetics of eosinophil depletion

The most immediate and objectively striking evidence of the biological effect of benralizumab was observed in the systemic inflammatory profile of the BANDOR cohort. At the time of enrollment (T0), the study population exhibited a profound T2 inflammatory signature, as evidenced by a median peripheral blood eosinophil count of 820 cells/µL (IQR: 515–1235). This baseline level of systemic eosinophilia reflects a state of high biological activity. Following the initiation of benralizumab, a rapid and profound cellular depletion was observed across all evaluation time points. The median absolute blood eosinophil count sharply declined from a baseline of 820 cells (IQR: 515–1235) to 0 cells (IQR: 0–10) at T3. This complete suppression of circulating eosinophils was stably maintained throughout the subsequent longitudinal follow-up, recording median values of 0 cells at T6, T12, and T18, respectively, confirming the durability of the biological response.

### 4.3 Clinical efficacy: evolution of asthma control, pulmonary function and oral corticosteroids intake reduction

The longitudinal evaluation of the lower airway compartment revealed a progressive improvement in both subjective symptom control and objective functional parameters over the 18 months observation period. At baseline (T0), the study population was characterized by poorly controlled asthma, with a median ACT score of 13 (IQR: 10–16.5), a value significantly below the universally recognized threshold of 20 for adequate clinical control.

Following the initiation of benralizumab, the cohort experienced a rapid and clinically meaningful shift in asthma control. By T3, the median ACT score had already risen to 22 (IQR: 19–24), indicating that the majority of patients transitioned from uncontrolled to well controlled status within the first trimester of therapy. This early symptomatic gain was not only maintained but showed a trend of further consolidation throughout the long-term follow-up. Specifically, median ACT scores were recorded at 23 (IQR: 18.5–24) at 6 months (T6) and 24 (IQR: 21–25) at 12 months (T12), ultimately stabilizing at a median of 23.5 (IQR: 20.2–25) at the 18 months mark (T18). The durability of this response is noteworthy, as it suggests that the nearly complete depletion of eosinophils translates into a sustained reduction of the inflammatory drivers responsible for bronchial symptoms.

In parallel with the improvement in patient reported outcomes, objective assessments of lung function via spirometry demonstrated a positive and stable trajectory. At baseline, the degree of bronchial obstruction was substantial, with a median FEV1 of 2.52L (IQR: 1.86–3.15), corresponding to a median of 65% of the predicted value (IQR: 50.5–80.5). During the treatment course, a gradual recovery of ventilatory function was observed. By T6, the median FEV1 had increased to 2.80L (IQR: 2.10–3.45), representing 75% of the predicted value. This functional improvement was preserved across the long-term observation window, with T18 measurements showing a median FEV1 of 2.85L and a predicted percentage of 77% (IQR: 62–87).

Regarding systemic corticosteroid sparing, 9 out of the 41 patients (22.0%) were on chronic maintenance oral corticosteroid therapy at baseline (TO), with a median daily prednisone-equivalent dose of 12.5 mg (IQR: 10.0–20.0 mg). Following the initiation of benralizumab, a progressive reduction in OCS requirements was observed. By the 18-month follow-up (T18), 7 of these 9 patients (77.8%) achieved complete OCS discontinuation, while the remaining 2 patients maintained a significantly lower daily dose (median 2.5 mg/day), demonstrating a robust steroid-sparing effect.

### 4.4 Impact on sinonasal burden: longitudinal analysis of SNOT-22 and its subdomains

At baseline (T0), the cohort demonstrated a severe sinonasal symptom burden, with a median total SNOT-22 score of 72 (IQR: 58.5–82.5). This baseline value reflects a population with severe disease, where the impact on quality of life was systemic. Following the initiation of benralizumab, the longitudinal analysis revealed a statistically significant and progressive decline in the total SNOT-22 score across all evaluation visits.

The most substantial clinical shift was observed during the early stages of treatment. By T3, the median total score had already dropped to 34 (IQR: 21–55), and this downward trajectory continued through the T6, reaching a median of 26 (IQR: 18–41.5). The achievement of the MCID, was reached by 73.2% of the cohort within initial 6 months’ time. The improvement remained stable during the long term follow-up, with median scores of 23 (IQR: 11.5–39) at 12 months and 22 (IQR: 12–37) at 18 months, suggesting that the therapeutic effect is early and not subject to significant waning or exhaustion over time.

To gain a more granular understanding of this response, a detailed dissection of the five specific SNOT-22 subdomains (Nasal, Ear/Facial, Sleep, Function, and Emotion) was performed. This analysis revealed that the anti-eosinophilic action of benralizumab provides a comprehensive benefit that transcends simple nasal patency:

- Nasal symptoms: this domain, encompassing core symptoms such as congestion, rhinorrhea and hyposmia/anosmia, showed the most rapid and pronounced response. From a high baseline median of 23 (IQR: 18.5–29.5), scores fell to 11 (IQR: 5.5–17) at T3, eventually stabilizing at a median of 7 by the end of the study. The rapid restoration of nasal airflow and the improvement in smell are likely direct consequences of the reduction in polyp volume and mucosal edema following eosinophil depletion.
- Sleep and Functional impact: at baseline, patients reported significant disruption in sleep architecture (median 13) and daily functional capacity (median 14). Both domains exhibited a synchronized and significant improvement by T6 (median 4 for sleep and 5 for function), with these gains maintained through T18. This suggests that the systemic control of T2 inflammation contributes to better nocturnal oxygenation and reduced daytime fatigue, which are critical components of the UAD burden.
- Ear/Facial and Emotional domains: while these domains presented with lower baseline scores (median 9 for Ear/Facial and 10 for Emotion), they nonetheless showed a consistent and statistically significant downward trend. By the T18 follow-up, median values had reached 2 and 3 respectively. The improvement in the emotional domain is particularly relevant, as it reflects a reduction in the psychological distress often associated with chronic sinonasal disease.

An interesting finding of the BANDOR study lies in the differential response kinetics observed across the various SNOT-22 subdomains. Rather than a uniform reduction in all symptoms, the data suggest that specific clusters of sinonasal manifestations are more highly sensitive to early IL-5Rα inhibition. This analysis provides clinicians with phenotypic red flags that may predict a high probability response to benralizumab.

The Nasal subdomain and the Ear/Facial subdomain emerged as the most rapid responders. At baseline, the Ear/Facial domain, although often overlooked in standard clinical assessments, was notably elevated in patients who later became early responders. Following the first three doses of therapy (T3), these specific clusters showed the fastest decline.

Interestingly, the data indicate that Sleep and Functional subdomains follow a slightly different trajectory. While these also showed significant improvement by T6, their recovery was more closely linked to the sustained stabilization of the lower airways.

These findings suggest that the baseline symptomatic distribution within the SNOT-22 questionnaire could serve as a phenotypic indicator of how the clinical response will unfold over time. In subjects where the initial disease burden is primarily driven by high Nasal and Ear/Facial scores, parameters often associated with acute mucosal edema and eosinophilic tissue infiltration (37), the data show that a significant portion of the total SNOT-22 reduction is achievable within the first 3 months of therapy.

### 4.5 Identification of baseline characteristics associated with early clinical response

In BANDOR study, patients were stratified based on their ability to reach the MCID for the SNOT-22 total score, defined as a reduction of at least 9 points, within the first 6 months of treatment This stratification resulted in two distinct comparative groups: early responders (n = 30, 73.2 percent), and late/suboptimal responders (n = 11, 26.8 percent), whose symptomatic recovery followed a more protracted trajectory. The median absolute eosinophil count in the early responder group was 885 cells/µL (IQR: 660–1420), whereas the late responder group presented with a substantially lower median of 530 cells/µL (IQR: 360–1100). This difference reached a high level of statistical significance (p < 0.001).

In addition to biological markers, the anamnestic history of the respiratory disease provided significant prognostic insights. The age at asthma onset emerged as a key variable, with early responders showing a significantly higher median age of 34 years (IQR: 25–40) compared to 26 years (IQR: 25–30) in the late responder group (p = 0.008). This distinction is particularly relevant when considering the UAD continuum.

Furthermore, the baseline functional status of the lower airways revealed an unexpected but statistically robust relationship with the sinonasal response. Early responders presented with a more severe degree of bronchial obstruction at T0, characterized by a median FEV1 of 57.5% of the predicted value (IQR: 44–70). In striking contrast, the late responder group exhibited a significantly higher baseline pulmonary function, with a median FEV1 of 79% (IQR: 60–90; p < 0.001). This inverse relationship, where higher baseline severity in the lower airways is associated with a more rapid achievement of the MCID in the upper airways, highlights the integrated nature of the respiratory system.

#### 4.5.1 Detailed baseline symptomatic profiles of early versus late responders

A more detailed investigation into the baseline characteristics of the study cohort allowed for the identification of specific symptomatic clusters that differentiate early responders from those with a more protracted recovery. By dissecting the SNOT-22 into its five constitutive subdomains at T0, we observed that the clinical signature of patients who reached the MCID within 6 months was primarily driven by a higher intensity of mechanical and physical sinonasal symptoms.

Similarly, the Nasal subdomain at baseline, which accounts for relevant symptoms such as nasal obstruction, discharge and the impairment of smell and taste, was notably more severe in the early responder group, with a median of 23 (IQR: 18–39). In comparison, late responders exhibited a median Nasal score of 17 (IQR: 12–22). While both subgroups suffered from significant sinonasal disease, the higher concentration of nasal specific symptoms in the early responders, points toward a phenotype where the disease burden is more localized and potentially more sensitive to the rapid depletion of circulating and tissue eosinophils.

The analysis also extended to the systemic and psychological impact of the disease, represented by the Sleep, Function, and Emotion subdomains. At baseline, early responders showed a high burden across these categories:

- Sleep domain: median of 14 (IQR: 10–16), encompassing symptoms like difficulty falling asleep and waking up at night.
- Function domain: median of 15 (IQR: 11–18), reflecting the impact on daily productivity and fatigue.
- Emotion domain: median of 11 (IQR: 8–14), indicating frustration, sadness or embarrassment related to the disease.

Interestingly, while these scores were high, they did not differ from the late responder group as the Ear/Facial and Nasal domains did.

## 5 Discussion

### 5.1 Summary of findings and the UAD perspective

The longitudinal data derived from the BANDOR study offer a robust characterization of the therapeutic trajectory in patients suffering from the complex intersection of SEA and CRSwNP. Over an 18 months observation period, the most relevant finding is the sustained and synchronized stabilization of the entire respiratory tract. This dual compartment recovery provides a compelling clinical confirmation of the UAD hypothesis, suggesting that when a shared inflammatory driver is effectively modulated, the clinical benefits transcend localized symptomatic relief to encompass a diffuse restoration of airway ease.

The cohort analyzed represented a particularly challenging Real-World population, characterized by significant baseline impairment, a high prevalence of comorbidities such as obesity and bronchiectasis, and a long-standing dependence on OCS. Despite these factors, the initiation of IL-5Rα targeted therapy induced a rapid transition from uncontrolled disease to a state of clinical stability. The fact that nearly three quarters of the patients achieved the SNOT-22 MCID within the first six months, while simultaneously showing significant gains in FEV1 and ACT scores, underscores the impact of addressing T2 inflammation at its systemic source. This synchronization is not merely a statistical correlation but reflects a physiological reset where the reduction of the eosinophilic burden in the blood translates into decreased mucosal edema and improved patency in both the bronchial tree and the paranasal sinuses.

One aspect of this study is the 18 months follow-up, which allows for a nuanced assessment of the durability of the therapeutic effect. In chronic respiratory management, a common concern is the potential for pharmacological exhaustion or the recurrence of symptoms during maintenance phases. However, results from BANDOR study demonstrate a remarkable stability in clinical outcomes over all the observation time, suggesting the biological depletion of eosinophils creates a stable environment that prevents the cyclical inflammatory flares typical of conventional steroid-based management.

A crucial point for interpreting our findings concerns the composition of the study cohort. By focusing exclusively on patients who completed the full 18 months observational period, we are inherently analyzing a per protocol population of clinical responders. In Real-World clinical practice, patients who experience a lack of efficacy or a suboptimal control of asthma symptoms, such as persistent exacerbations or failure to reduce oral corticosteroid dependence, are significantly more likely to discontinue the biologic agent or be switched to a different treatment during the first months of therapy.

Therefore, it must be acknowledged that our data reflect the outcomes of individuals who achieved a satisfactory clinical response, at least regarding their SEA. This creates a specific clinical context: we are observing the long term evolution of CRSwNP specifically within a subgroup where benralizumab successfully managed the lower airway inflammation. Consequently, the significant improvements observed in SNOT-22 and its subdomains scores and should be viewed as the potential sinonasal benefit achievable when the drug is effective enough to ensure long term treatment adherence. While this confirms the strength of the UAD approach, it also suggests that the reported efficacy might be higher than what would be observed in an intention to treat analysis that includes early dropouts or non-responders.

The UAD perspective adopted in this analysis highlights the necessity of a multidisciplinary approach to severe T2 disease. The baseline profile of our patients, marked by severe sinonasal obstruction (SNOT-22 median of 72) and significant airflow limitation, illustrates how these conditions exacerbate one another, creating a feedback loop of respiratory distress. The evidence that a single systemic intervention can interrupt this cycle provides a strong rationale for integrating pulmonary, allergologic/immunollogic and ENT evaluations into a unified diagnostic and therapeutic pathway.

### 5.2 The biological foundation: permanent eosinophil depletion

The most definitive biological marker of treatment efficacy in the BANDOR cohort was the rapid and sustained exhaustion of peripheral blood eosinophils. At the baseline assessment, the study population exhibited a median count of 820 cells/µL, a value that signifies a state of intense Type 2 systemic inflammation despite the ongoing use of high-dose inhaled or oral corticosteroids. The transition from this baseline hypereosinophilia to a consistent median of 0 cells/µL by the third month of therapy represents a relevant shift in the cellular composition of the respiratory tract. Unlike other biotechnological drugs that target the IL-5 ligand, which typically lead to a partial reduction in eosinophil counts, the mechanism of action observed results in an almost total clearance of these effector cells from the circulation.

The precision of this mechanism is reflected in the longitudinal stability of the data; the stable depletion recorded from the third to the eighteenth month suggests that the bone marrow output and the peripheral survival of eosinophils are effectively neutralized. A complete and durable suppression is critical in the context of UAD, where the persistent infiltration of eosinophils into the bronchial and sinonasal mucosa is the primary driver of tissue remodeling, epithelial barrier disruption and polyp formation (38–41), by switching off release of major basic protein, eosinophil-derived neurotoxin and leukotrienes, the treatment effectively silences the destructive inflammatory cascade (42–45). This biological reset appears to allow for a period of mucosal healing and stabilization that is difficult to achieve when eosinophils remain present, even at reduced levels (46, 47).

While other biomarkers, such as FeNO or total serum IgE, may be influenced by multiple overlapping pathways or atopic status, the sustained suppression of peripheral eosinophils over 18 months remains a direct indicator of the treatment’s primary pharmacological effect. The evidence presented in this selected cohort confirms that the systematic and persistent elimination of these cells is a fundamental milestone in the management of severe T2 disease, providing the foundation for the long term clinical remission observed in both the upper and lower respiratory compartments.

### 5.3 Lung function and asthma control: beyond clinical trials

The longitudinal progression of pulmonary function and asthma control within the BANDOR cohort provides a contribution to the understanding of IL-5Rα inhibition in a clinical condition characterized by high phenotypic complexity. At the time of enrollment, the study population presented with a marked degree of bronchial impairment, as reflected by a median FEV1 of 65% of the predicted value and an ACT score of 13. These baseline parameters are indicative of a population with SEA, where conventional high dose inhaled therapies and long acting bronchodilators had failed to achieve clinical stability. The subsequent transition to a median FEV1 of 77% and an ACT score of 23.5 at the T18 suggests that the systematic removal of the eosinophilic driver facilitates a profound functional recovery that is both rapid in onset and durable over an extended observation period.

When these results are contextualized alongside pivotal Phase III registration trials, such as SIROCCO and CALIMA (48–50), several observations emerge regarding the magnitude of the functional gain. While RCTs reported a mean FEV1 improvement ranging between 120 mL and 150 mL, the BANDOR cohort exhibited a median increase from 2.52L to 2.85L (330mL). This functional response observed in our Real-World setting is likely linked to the high baseline inflammatory intensity of the participants. However, it must be emphasized that these results specifically characterize a subset of persistent responders who remained on benralizumab for the full 18 months period. In a clinical practice scenario, patients exhibiting suboptimal functional or symptomatic recovery would have been discontinued or switched to alternative biologic classes; thus, the link between baseline inflammation and functional gain should be interpreted as a hallmark of successful long term treatment maintenance in appropriately selected subjects (46–49, 51–54).

### 5.4 Impact on sinonasal burden and quality of life: a multidimensional analysis

The baseline data revealed a population in a state of profound clinical distress, with a median total SNOT-22 score of 72. This value represents a level of impairment that transcends simple nasal obstruction, indicating a significant compromise of overall quality of life. The achievement of the MCID in 73.2% of the cohort within the first six months is a critical finding, as it confirms that the biological suppression of eosinophils translates directly into a perceptible clinical benefit for a vast majority of patients who had previously failed conventional management.

The granularity of the response becomes evident when examining the individual subdomains of the SNOT-22. The Nasal symptoms domain, which encompasses the most direct manifestations of nasal polyposis such as congestion and hyposmia, showed the most immediate and pronounced reduction. The decrease from a median of 23 at baseline to 11 at three months, eventually stabilizing at a median of 7, suggests that the depletion of circulating eosinophils leads to a rapid resolution of sinonasal symptom burden, though objective endoscopic assessments of polyp volume were not systematically captured. This early recovery of nasal patency is essential, as it addresses the mechanical drivers of the disease that are often the most frustrating for patients. However, the comprehensive nature of the treatment is further illustrated by the significant improvements in the Sleep and Functional subdomains. The baseline scores for sleep disruption (median 13) and functional limitation (median 14) underscore the systemic reach of CRSwNP. The restoration of normal sleep and daily productivity, which reached their maximal stability already by at sixth months, indicates that the therapeutic effect extends beyond the sinonasal cavities to improve global physiological wellbeing.

A significant point of discussion is the durability of the sinonasal response throughout the 18 months of follow-up. In many cases of severe CRSwNP, patients experience a cyclical pattern of improvement followed by relapses, particularly following the tapering of OCS or after surgical interventions. The plateau in SNOT-22 scores observed between the 12th and 18th months in our cohort indicates that, for those patients who respond and remain on treatment, the continuous exhaustion of the eosinophilic driver effectively prevents the typical recurrence of inflammatory tissue. While this stability must be interpreted within the context of a maintenance biased population, where only those achieving clinical benefit persisted continued therapy, it nevertheless provides a strong rationale for IL-5Rα targeting as a long term maintenance strategy in these phenotypes. By sustaining clinical remission in the upper airways for those who tolerate and respond to the drug, benralizumab potentially reduces the cumulative necessity for repeated sinus surgeries and chronic corticosteroid exposure, both of which are associated with significant long term morbidity.

Furthermore, the synchronization of the SNOT-22 improvements with the gains in ACT and pulmonary function (FEV1) reinforces the integrated management of UAD. The data suggest that the emotional domain, which also showed a statistically significant downward trend from a median of 10 to 3, reflects the psychological relief associated with the resolution of chronic overall respiratory distress. The reduction in frustration, sadness and embarrassment reported by the patients indicates that the successful management of the biological inflammatory driver has a secondary but equally important impact on mental health. This multifaceted recovery underscores the necessity for clinicians to move beyond a localized anatomical focus. The ability of a single systemic intervention to provide an improvement across physical, functional and emotional domains confirms that addressing the common eosinophilic pathway is a highly effective strategy for restoring the quality of life in patients burdened by the complex interplay of upper and lower airway disease.

The heterogeneous response rates observed among the five SNOT-22 subdomains warrant a detailed pathophysiological interrogation, as they provide critical insights into the resolution patterns of UAD.

The immediate relief observed in the Nasal and Ear/Facial domains (characterized by a median reduction of over 50% by T3) is likely attributable to the direct impact of IL-5Rα inhibition on acute inflammatory parameters. These subdomains predominantly reflect mechanical and obstructive symptoms, such as nasal congestion, rhinorrhea, ear fullness and facial pressure, which are driven by mucosal edema, vascular permeability and the consequent rapid recruitment of eosinophils into sinonasal tissue. By silencing the release of eosinophil derived granule proteins and leukotrienes (1, 30, 42), the treatment seems to facilitate an early reduction in mucosal swelling and a potential retraction of polypoid volume, thereby providing rapid relief from the physical pressure that characterizes the most severe baseline presentations.

Conversely, the slower kinetic of recovery recorded in Sleep, Functional and Emotional subdomains suggests that these parameters are influenced by factors beyond acute mechanical obstruction. While sleep disruption is undeniably linked to nasal patency, the restoration of high quality sleep, reflected in the SNOT-22 Sleep domain, may require a more sustained period of systemic stabilization. Chronic inflammation in severe eosinophilic asthma and CRSwNP induces a state of persistent neurobiological and autonomic dysregulation (55); consequently, the biological reset achieved through eosinophil depletion may necessitate several months to translate into improved nocturnal oxygenation and the normalization of sleep cycles.

Furthermore, Functional and Emotional subdomains (encompassing parameters such as fatigue, reduced productivity, frustration and sadness) are tied to the long term psychological burden of living with a chronic disease (56–58). These domains represent the cumulative impact of years of poorly controlled symptoms and frequent corticosteroid intake. The data suggest that even after the physical airway obstruction has been resolved, a period of clinical consolidation is required for the patient to perceive a complete restoration of daily functionality and emotional wellbeing. This resolution, where the mechanical obstruction is cleared first, followed by the systemic and psychological recovery, highlights the importance of the 18 months longitudinal perspective. It suggests that a short term evaluation might underestimate the total therapeutic value of the intervention if the assessment is limited to aggregate scores without considering the nuanced recovery of these non-physical domains.

In contrast to IL-4/IL-13 inhibition, which has demonstrated an exceptionally rapid reduction in SNOT-22 scores, likely due to the immediate stabilization of the epithelial barrier and reduction of mucosal edema, the clinical trajectory of benralizumab in the BANDOR cohort suggests a two phases recovery. While the depletion of peripheral eosinophils is nearly instantaneous, the subsequent clinical benefit unfolds through an early improvement in physical domains (Nasal and Ear/Facial), followed by a more gradual consolidation of functional and emotional domains. This priming period represents the time necessary for tissue remodeling and the resolution of chronic systemic fatigue following the total exhaustion of the eosinophilic driver. Data at 18 months confirm that, for persistent responders, this cellular targeted approach provides a stability of remission that is highly competitive with the benchmarks set by other biologic classes, especially in patients with high levels of peripheral eosinophilia.

### 5.5 Predictors of clinical response: deciphering the early responder phenotype

The identification of baseline clinical and biological markers capable of predicting the velocity of therapeutic success represents a fundamental step toward the implementation of precision medicine in UAD. By stratifying the cohort into early and late responders based on the achievement of the SNOT-22 MCID at six months, several statistically significant divergences emerge, providing a multifaceted profile of the patient most likely to experience a rapid symptomatic recovery. The data indicate that the ideal early responder is characterized by a high systemic inflammatory load, a more severe degree of baseline bronchial obstruction and an adult-onset asthma phenotype, as well as a specific symptomatic signature dominated by mechanical sinonasal distress (**Figure 1**).

**Figure 1:**
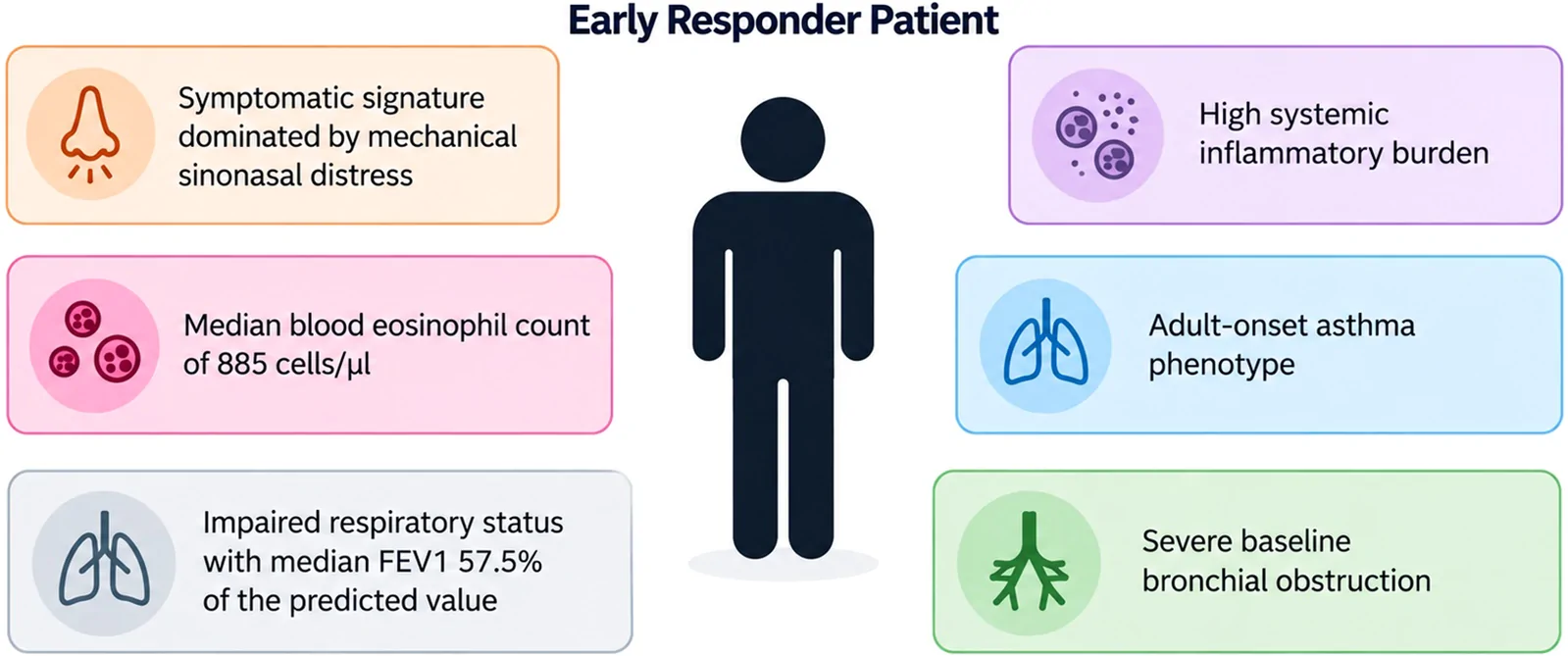
Baseline clinical, biological, and functional signature of the early responder phenotype in the BANDOR study. The schematic outlines the key baseline characteristics that identify patients achieving a rapid and meaningful therapeutic response (SNOT-22 MCID reduction ≥ 9 points within the first 6 months of benralizumab therapy): (1) a high systemic inflammatory burden and elevated baseline peripheral blood eosinophil count (median 885 cells/µL); (2) an adult-onset asthma phenotype; (3) a severe baseline bronchial obstruction with a median FEV1 of 57.5% of the predicted value; and (4) a predominant symptomatic signature characterized by mechanical and physical sinonasal distress (elevated Nasal and Ear/Facial subdomains).

The role of peripheral blood eosinophilia as an associated baseline features of response timing is particularly robust. In these cases, the rapid reduction of the cellular infiltrate (59) leads to a precipitous reduction in mucosal edema, facilitating a faster resolution of the inflammatory process compared to patients with lower eosinophilic levels and perhaps more remodeled tissues.

A seemingly paradoxical finding concerns the relationship between baseline pulmonary function and sinonasal recovery. Early responders presented with a significantly more impaired respiratory status at T0, showing a median FEV1 of 57.5% of the predicted value, whereas late responders had a substantially higher baseline FEV1 of 79% (p < 0.001). This inverse correlation highlights the integrated nature of the respiratory system. Patients with the greatest functional impairment across the entire UAD spectrum may be the most sensitive to the systematic depletion of eosiniphils in this cohort. In these patients, the perceived benefit measured through the SNOT-22 is likely amplified by the simultaneous relief of severe bronchial obstruction. The synchronization of the recovery across both compartments suggests that high baseline severity does not act as a barrier to efficacy but rather as an indicator of a phenotype that is dependent on the eosinophilic pathway, and thus highly responsive to its targeted removal.

Furthermore, the timing of respiratory disease onset provides additional prognostic insights. The early responder group was characterized by a significantly higher median age at asthma onset (34 years) compared to the late responder group (26 years; p=0.008). This observation supports the hypothesis that adult onset eosinophilic asthma represents a distinct clinical entity, often characterized by a more explosive and pure T2 inflammation. Conversely, an earlier onset of respiratory symptoms is frequently associated with a longer duration of chronic inflammation, which may lead to irreversible tissue remodeling, fixed anatomical changes in the paranasal sinuses or the development of overlapping inflammatory pathways (such as neurogenic inflammation or dysbiosis) (60–68). In such cases, the symptomatic recovery following biological therapy may follow a more protracted trajectory, as the pharmacological intervention must overcome years of structural damage and chronic mucosal alterations.

The analysis of baseline SNOT-22 subdomains further refines this exploratory associative framework. The significantly higher Ear/Facial and Nasal scores observed at baseline in early responders suggest that a symptomatic profile dominated by pressure and obstruction is a red flag for rapid clinical success. These mechanical symptoms may be the direct consequence of eosinophilic tissue infiltration and acute edema, both of which are highly reversible through IL-5Rα inhibition. Conversely, when the total SNOT-22 score is heavily influenced by Sleep or Emotional domains at baseline, without a corresponding high intensity of physical/mechanical symptoms, the recovery appears to be slower. This detailed reporting of the baseline clinical correlates confirms that the early responder is not merely a patient who improves faster, but one who possesses a specific inflammatory characteristic a patient that is the optimal candidate for the rapid biological reset offered by this therapeutic strategy.

### 5.6 Clinical implications and precision medicine

The identification of distinct symptomatic and biological response trajectories suggests that the initial assessment should move beyond the mere documentation of disease severity to encompass a detailed phenotyping of the individual inflammatory signature. The finding that a baseline profile characterized by high systemic eosinophilia and a symptomatic burden dominated by mechanical sinonasal distress acts as a reliable indicator of rapid clinical benefit offers a significant advantage in the allocation of therapeutic resources. In a clinical panorama where multiple biological options are available, the ability to match the specific mechanism of IL-5Rα inhibition to the patients most likely to experience a rapid symptomatic improvement is a fundamental step toward optimizing long term outcomes and cost effectiveness.

From a practical perspective, the differentiation between early and late responders allows for a more nuanced approach to patient counseling and follow-up scheduling. For individuals who align with the early responder phenotype, the rapid achievement of the MCID within the first three to six months serves as a crucial biological validation of the intervention’s efficacy. In these cases, the specialist can communicate an optimistic prognosis regarding the speed of recovery, which significantly enhances patient trust and treatment adherence. Conversely, for patients whose baseline profile suggests a more protracted response, perhaps due to a longer history of disease, lower eosinophilic drive or a symptomatic burden more heavily weighted toward sleep and emotional dysfunction, the data provide a rationale for maintaining the treatment beyond the initial induction phase. This prevents the premature assessment of therapeutic failure and encourages persistence during the months required for systemic and functional stabilization to manifest. It is essential to recognize that a lack of immediate mechanical relief does not equate to biological non responsiveness but rather reflects a different kinetic of mucosal response.

Nevertheless, for BANDOR persistent cohort, the suppression of the eosinophilic driver appears to induce a functional reset, potentially altering the natural history of their respiratory disease. By providing a durable alternative to the cyclical use of systemic corticosteroids, this approach directly addresses the cumulative morbidity of traditional management, a point of particular relevance given that 22% of the cohort had already experienced OCS-related adverse events at baseline. The prevention of inflammatory rebound in these subjects suggest that, when the initial response is favorable, the systemic modulation of T2 inflammation serves as a cornerstone for protecting the patient’s long term respiratory reserve.

A further implication of these findings concerns the optimization of surgical timing in patients with comorbid nasal polyposis. The ability of IL-5Rα targeted therapy to induce a rapid reduction in the Nasal and Ear/Facial subdomains suggests that biological treatment could potentially reduce symptom severity and the need for repeated interventions, though prospective trials incorporating objective nasal polyp scores and formal olfactory testing are required to confirm structural modifications with a concomitant clinical benefit across upper and lower respiratory domains, reducing the need for invasive procedures or improving the surgical field by minimizing mucosal inflammation and bleeding. In the era of precision medicine, the integration of detailed SNOT-22 subdomain analysis into the surgical decision making process allows for a more precise selection of candidates who might benefit more from pharmacological stabilization than from immediate anatomical correction. This shift toward a biologic first or biologic integrated approach in severe relapsing cases represents a significant evolution in the standards of care for chronic rhinosinusitis.

Ultimately, the clinical implications of the BANDOR study reside in the shift toward a more granular interpretation of patient reported outcomes. Utilizing the SNOT-22 as a multidimensional map rather than a simple aggregate score enables a more precise analysis of the response kinetics. This level of detail is essential for the implementation of precision medicine, as it allows clinicians to anticipate the resolution patterns of different symptomatic clusters. By integrating biological markers with detailed symptomatic profiling, the specialist can move toward a highly personalized management plan, ensuring that the timing of clinical interventions and assessments is aligned with the expected biological and functional recovery of each single patient. This approach not only optimizes clinical success but also redefines the patient’s journey from chronic disease management to a sustained state of respiratory health and well-being (**Figure 2**).

**Figure 2:**
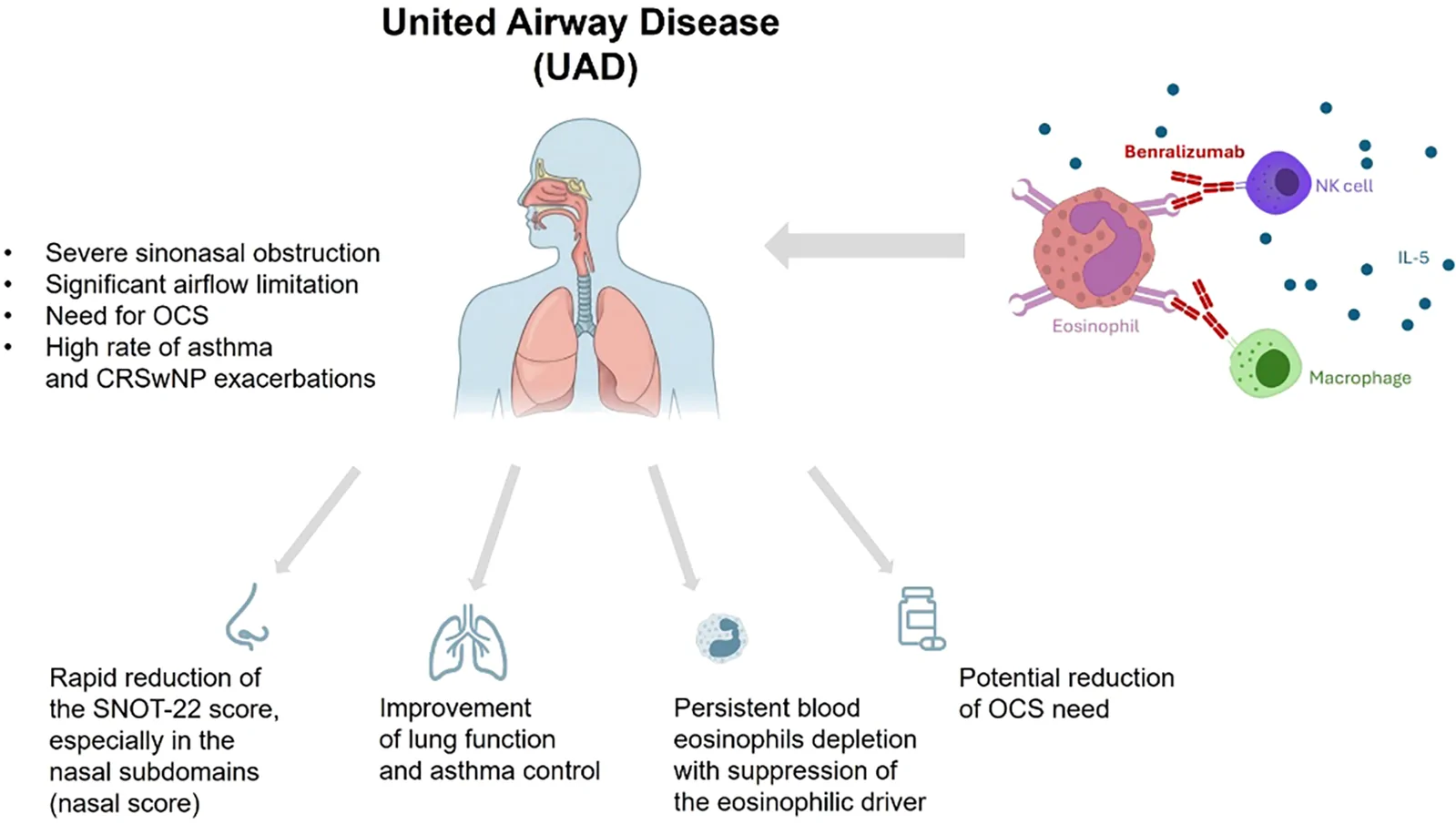
Multidimensional therapeutic framework of benralizumab in the United Airways Disease (UAD) model. The schematic illustrates the systemic and compartmental clinical impacts of targeted IL-5Ralpha inhibition over an 18-month observation period in a per-protocol maintenance cohort of severe eosinophilic asthma (SEA) and chronic rhinosinusitis with nasal polyps (CRSwNP) patients. (Top Right) Mechanism of action showing benralizumab-mediated antibody-dependent cell-mediated cytotoxicity (ADCC) via NK cells, leading to a profound, rapid, and sustained depletion of systemic and tissue eosinophils. (Center/Left) Baseline clinical phenotype characterized by severe sinonasal obstruction, significant lower airway limitation, high systemic inflammatory burden, and oral corticosteroid (OCS) dependency. (Bottom) Longitudinal downstream therapeutic consequences reflecting a dual-compartment recovery cascade: (1) rapid early reduction in patient-reported sinonasal symptom scores (SNOT-22), predominantly driven by physical and nasal subdomains; (2) sustained long-term improvement in pulmonary function (FEV1) and asthma control (ACT); (3) persistent biological suppression of the eosinophilic driver; and (4) a robust steroid-sparing effect with complete OCS weaning in the majority of dependent patients.

## 6 Strengths and limitations

The clinical and biological insights provided by the BANDOR study must be interpreted within the framework of its specific methodological strengths and limitations. A primary strength of this investigation is the 18 months longitudinal follow-up, essential for characterizing the long term stability of the respiratory tract and for confirming that the biological depletion of eosinophils does not exhibit a waning effect over time. Moreover, the dissection of the SNOT-22 into its constitutive subdomains represents a significant methodological advantage. By moving beyond the aggregate score, it was possible to identify differential recovery kinetics between mechanical obstructive symptoms and systemic or emotional domains, providing a more complex map of the patient’s journey toward clinical remission.

However, several limitations warrant careful consideration. The first concerns the sample size, which was restricted to 41 participants. While the statistical significance of the primary and secondary outcomes remained robust, the relatively small cohort may limit the capacity to perform complex subgroup analyses or to generalize the findings to a broader population.

Given the limited sample size (n = 41), findings regarding baseline factors associated with early response are derived from exploratory comparative analyses without multivariable adjustment; therefore, terms such as ‘predictor’ or ‘determinant’ should be interpreted with caution, and future larger prospective studies are warranted to confirm these associations.

Additionally, the study population was exclusively composed of patients resident in the Italian territory treated within a specific national healthcare framework. This geographical and ethnic homogeneity, while ensuring consistency in standard of care background therapies, implies that the results should be validated in larger multiethnic cohorts to account for potential genetic, environmental, or dietary influences on the T2 inflammatory landscape.

A critical contextual limitation resides in the retrospective nature of the study and the specific clinical indication for the treatment. All participants in the BANDOR cohort suffered from comorbid SEA, which served as the primary medical justification for the initiation of IL-5Rα targeted therapy. The therapeutic agent does not possess a formal regulatory indication for the treatment of CRSwNP as a standalone condition. Consequently, the observed sinonasal benefits occurred in a Real-World population where the upper airway pathology was inherently linked to severe lower airway disease. This asthma-first recruitment strategy means that the results may not be entirely representative of patients with isolated nasal polyposis or those with milder or non-asthmatic respiratory phenotypes.

A major limitation of the present study, which must be carefully considered when interpreting its findings, is the inherent selection bias, specifically a survivorship bias, stemming from the 18-month observation period. By including only patients who completed the full course of continuous treatment, our analysis is by definition restricted to a persistent responder population, thereby excluding early treatment failures, absolute non-responders, and those who switched therapies during the initial months. Among the 54 initially screened patients, 13 discontinued treatment or were excluded prior to 18 months (6 for early lack of efficacy, 4 due to loss to follow-up, and 3 for adverse events/other reasons). By including only the 41 patients who completed the full course of treatment, our analysis is by definition restricted to a persistent responder population. Consequently, the high rates of clinical and functional success reported here likely represent an overestimation of the drug’s effectiveness in an unselected intention to treat population. While these data provide a valuable blueprint of the long term clinical trajectory and the reset potential of Benralizumab in successful cases, they cannot be extrapolated to predict the initial response rate across the broader and more heterogeneous spectrum of all patients starting the therapy.

Furthermore, the absence of a placebo control group, a common feature of retrospective Real-World evidence, must be acknowledged. The subjective improvements in SNOT-22 scores could theoretically be influenced by the psychological impact of receiving a high technology biological intervention and its effects on asthma. Nevertheless, the high degree of synchronization between objective functional recovery and patient reported outcomes, particularly the selective rapid decline of the Nasal and Ear/Facial subdomains, suggests that the perceived benefits are fundamentally rooted in the systematic suppression of the eosinophilic driver rather than in a nonspecific placebo response.

In addition, by categorizing patients into early versus late/suboptimal responders, we recognize that subtle differences between delayed recovery and minimal clinical response may warrant further investigation in larger prospective cohorts.

Finally, the retrospective design implies that objective sinonasal evaluations, such as standardized nasal polyp scores (NPS) or formal olfactory testing, were not systematically available across all time points, limiting our sinonasal efficacy assessment primarily to patient-reported outcome measures (SNOT-22).

## 7 Conclusions

The BANDOR study provides evidence of the efficacy and durability of IL-5Rα targeted therapy in a Real-World cohort of patients suffering from the complex intersection of SEA and CRSwNP. Over an 18 months observation period, the systematic depletion of circulating eosinophils led to sustained clinical improvement in patient-reported sinonasal symptoms alongside asthma control. This dual compartment recovery reinforces the clinical validity of the UAD framework, demonstrating that addressing the common inflammatory driver at its systemic source is a highly effective strategy for managing the most recalcitrant Type 2 phenotypes.

A contribution of this research is the identification of specific clinical characteristics that may predict a rapid therapeutic response. The evidence indicates that patients with adult onset disease, high baseline systemic eosinophilia and a symptomatic burden dominated by mechanical sinonasal distress, particularly in the Nasal and Ear/Facial subdomains, are the most likely to achieve significant clinical benefit within the first six months of treatment. Conversely, the more gradual recovery observed in the Sleep, Functional and Emotional domains highlights the necessity of a long term therapeutic perspective to achieve full clinical remission. These findings allow for a transition toward a more personalized management model, where the baseline symptomatic profile serves as a data driven guide for patient counseling and follow-up scheduling.

Furthermore, the long term functional stability and the high rate of achievement of the MCID of SNOT-22 observed in this cohort stand in contrast to the more variable results reported in previous registration trials. This discrepancy underscores the importance of patient selection and the relevance of Real-World evidence in identifying the high responder populations that may have been diluted in more heterogeneous randomized controlled trials.

In conclusion, the results of the BANDOR study support the early integration of biological therapies into the multidisciplinary management of UAD. The ability to induce a durable state of mucosal healing and to protect the functional reserve of the entire respiratory tract represents a fundamental milestone in the evolution of respiratory medicine. Future research should aim to validate these predictive markers in larger multicenter cohorts, further refining the criteria for the optimal selection of biological interventions and ensuring that the promise of precision medicine is fully realized for all patients burdened by severe eosinophilic airway disease. Also, future prospective studies incorporating objective endoscopic and olfactory evaluations are warranted to confirm structural mucosal changes.

## Data Availability

The raw data supporting the conclusions of this article will be made available by the authors, without undue reservation.
